# Can you hear me now? Momentary increase in smartphone usage enhances neural processing of task-irrelevant sound tones

**DOI:** 10.1016/j.ynirp.2022.100131

**Published:** 2022-09-13

**Authors:** Mark van de Ruit, Arko Ghosh

**Affiliations:** a Department of Neurology, Leiden University Medical Center, Leiden, the Netherlands; b Department of Biomechanical Engineering, Delft University of Technology, the Netherlands; c Cognitive Psychology Unit, Institute of Psychology, Leiden University, the Netherlands

## Abstract

According to popular belief when engaged on the smartphone surrounding information is ignored. However, emerging ideas based on laboratory-designed tasks suggest that the processing of task-irrelevant (distractor) information is enhanced when cognitive load is high as anticipated during intense periods of smartphone usage. Here we address the neural processing of task-irrelevant auditory tones while interacting with the smartphone touchscreen. We analyzed neural activity (EEG) while people (N = 24) were seated in public spaces and used their smartphones for ∼1.5 h. During this period, the number of touchscreen interactions spontaneously varied from one moment to another. The central and frontal theta-band (4–8 Hz) oscillations, an index of cognitive load, increased proportionally to the number of interactions. Moreover, an index of excitation:inhibition balance derived from the aperiodic signal components increased with the interactions. The auditory tones resulted in prominent evoked potentials with peaks at ∼50 ms, ∼100 ms, and ∼200 ms, reflecting the different cortical information processing stages. Of these, the ∼100 ms component was specifically related to the number of interactions such that the higher the number of interactions, the larger the neural signal amplitudes. Contrary to the popular notions but in keeping with emerging ideas on cognitive load, auditory information processing is enhanced with increased smartphone usage. In daily life, neural processing of the surroundings is partly shaped by the immediate cognitive demands imposed by the smartphone.

## 1 Introduction

Smartphones are routinely used to perform meaningful actions, and how smartphone-irrelevant (i.e., distractor) information is processed by the brain is important to elucidate. According to a held view – prevalent in both common discourses and behavioral sciences – the more engaged on the smartphone, the harder it is to process a non-smartphone information source (Larue et al., 2020; Lin and Huang, 2017). Superficially, these ideas borrow from the mechanistic accounts of attention that suggest behavior (say smartphone interactions) is supported by the reactive or pro-active suppression of distractor information (non-smartphone information) (Geng, 2014).

Laboratory-designed tasks provide an alternative account of how distractors are processed when deeply engaged in a task. According to emerging behavioral evidence, spanning different task types and sensory modalities, tasks with high cognitive load enhance distractor processing (Dalton et al., 2009; Lavie, 2010; Lavie et al., 2014; Macdonald and Lavie, 2011). Moreover, the interaction between auditory and visual processing is enhanced with a high cognitive load (Michail et al., 2021; Michail and Keil, 2018). According to the *cognitive* load theory, demanding tasks occupy frontal cortical functions thereby limiting their ability to prevent distractor processing. The stages of information processing impacted by the cognitive load can be revealed using the measurement of brain activity (electroencephalography – EEG). For instance, when engaged in a visual working memory task, auditory evoked potential components that reflect primary processing are enhanced (Regenbogen et al., 2012). Note, that a distinct pattern of results forms with increased *perceptual* load and the distractor presented in the same modality – such that the higher the perceptual load the lower the distractor processing (Lavie et al., 2014). It is unclear if and how these ideas apply to real-world behavior. This study shall address: Does engaging on the smartphone enhance the processing of surrounding information?

A challenge posed by the dynamic and complex nature of real-world behavior is to establish the momentary cognitive demand imposed by the behavior. This is in contrast to the carefully designed laboratory tasks where the cognitive load can be specifically manipulated. Intuitively, active smartphone behaviors requiring frequent user inputs are more cognitively demanding than the more passive behaviors conducted on the phone. In this real-world behavioral setting, a generic EEG marker of cognitive load – already well-established in controlled laboratory settings – can be leveraged. In particular, neural theta oscillations in EEG signals are considered a measure of cognitive load such that the higher the load, the larger the power in the theta band (Gevins and Smith, 2000; Kahana et al., 1999).

In laboratory tasks, attentional processes are associated with a modality-specific increase in excitation in the sensory circuits (Waschke et al., 2021). Here the authors leveraged recent computational tools to infer the neural excitation:inhibition balance over the central (targeting auditory circuits) and occipital electrodes (targeting visual circuits) based on the aperiodic components (the fitted exponent and the shift) of the power spectral density (Gao et al., 2017; Jacob et al., 2021). The smaller the exponent (smaller the absolute value, the shallower power spectral density) the larger the ratio (i.e., more excitation). Notably, when in a vigilant state the exponent is smaller – indicating more neural excitation – than in unconscious states such as in sleep or under anesthesia (Colombo et al., 2019). The *offset* – capturing the broadband power – of the aperiodic fit reflects the neural spiking activity (Manning et al., 2009; Miller et al., 2009). Interestingly, with reduced arousal (as in under anesthesia), the offset increases (Lendner et al., 2020). Moreover, the lower the offset the larger the hemodynamic activity in the prefrontal cortical networks engaged in executive control (Jacob et al., 2021). Our observations will help reveal how these parameters vary when engaged on the smartphone. Are the periods of intense smartphone interactions accompanied by a general increase in neural excitability and diminished neural spiking – as in a vigilant or aroused state?

In this study, we recorded EEG signals while participants engaged on their smartphone touchscreen in designated public spaces (campus café and seating area) for ∼1.5 h. By using a background app we quantified the touchscreen interactions. We probed auditory processing by using smartphone-unrelated tones intermittently presented throughout the entire recording period. Our analysis suggests that in periods of heightened smartphone usage, cognitive load increases, accompanied by increased neural excitation diminished neural spiking activity, and enhanced cortical processing of auditory information.

## 2 Results

### 2.1 Neural oscillations related to smartphone behavior

**Fig. 1:**
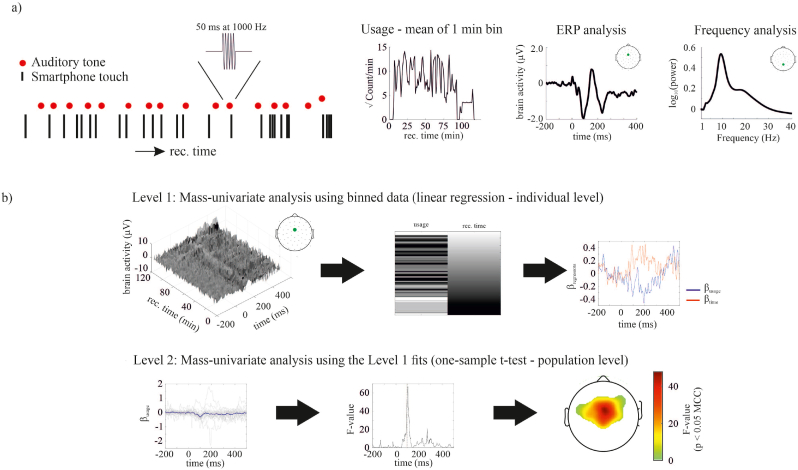
A mobile EEG setup to study the processing of auditory tones while using the smartphone. (a) While participants interacted with their smartphones, an auditory tone was played to both ears every ∼1–3 s (red dots). Each vertical black dash representant a single smartphone touch. Inter-touch intervals range from 100 ms to 5 s. An example time-series of smartphone use throughout an experiment. Smartphone use is quantified as the square root of the number of touches accumulated in a 1-min bin. The EEG was analyzed to study changes in the auditory evoked potentials and spectral properties for each participant and recorded channel separately. (b) The ERPs and periodogram were extracted in 1 min bins and used in a mass univariate regression with the factors of smartphone usage and time spent on the phone (level 1). Subsequently, one-sample t-tests were performed to confirm consistency of β-parameters across participants and channels (level 2). (For interpretation of the references to colour in this figure legend, the reader is referred to the Web version of this article.)

The number of smartphone interactions spontaneously generated by the participant varied from one moment to the next (Fig. 1). We related this behavioral variability to the EEG signals at the level of each participant using mass univariate regressions and then conducted follow-up statistics using the regression slopes to reveal the patterns consistent across the sample. The regressions considered the recording duration as an explanatory variable in addition to the number of interactions. We ran separate regression analyses focused on the EEG spectral properties, the aperiodic components, and the auditory event-related potentials.

**Fig. 2:**
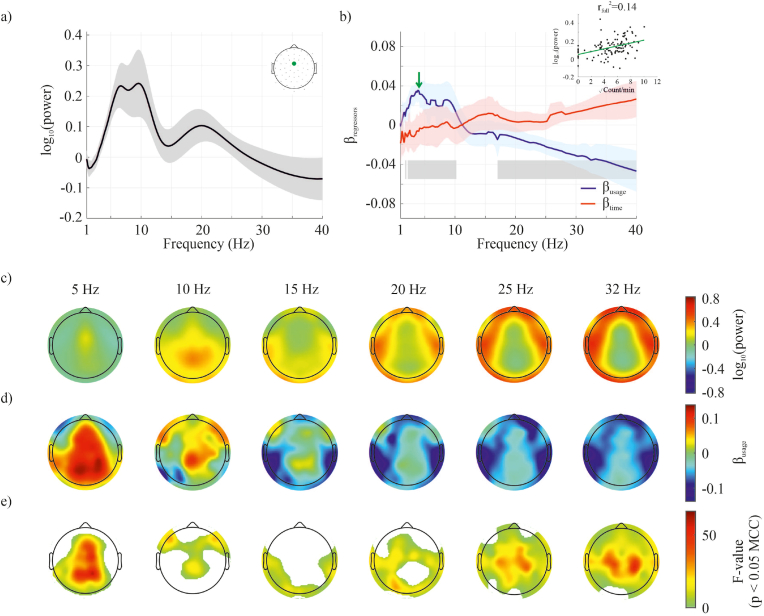
EEG power spectrum is related with smartphone usage and the time spent on the phone. The relationships were observed at the level of each subject using linear regressions, and we performed one-sample t-tests of the regression coefficients (*β*) stemming from each subject. An increase in central and frontal theta band power (4–8 Hz) is observed with increased smartphone usage (a) The FOOOF corrected power spectrum (mean) and corresponding 95% confidence interval are shown for a central electrode (green dot). (b) The corresponding *β-*values (mean and 95% confidence interval) were derived from the regression model for the factors smartphone usage (i.e., number of interactions, blue line) and time spent on the phone (red line). The frequencies where significant statistical clusters related to smartphone usage were found (according to the one-sample *t*-test corrected for multiple comparison correction, MCC) are shaded in grey. The inset shows the regression model's *β-*value at a single frequency (for visualization). No significant effects for the factor time were found (see Supplementary Fig. 1). (c) Scalp plots show the mean (FOOOF corrected) power for specific frequencies across the power spectrum. (d) Corresponding β-values of the regression model for the factor smartphone usage. (e) Corresponding F-values of the regression model for the factor smartphone usage masked for significance after MCC (note, F = T^2^ with the T value stemming from the one sample *t*-test). (For interpretation of the references to colour in this figure legend, the reader is referred to the Web version of this article.)

The spectral analysis (periodograms) was adjusted for aperiodic components (Donoghue et al., 2020). The topology of the population average power revealed theta band (4–7 Hz) oscillations more prominently at the central & frontal electrodes, the alpha band (8–12 Hz) oscillations at the occipital electrodes, and the beta band (13–30 Hz) bilaterally over the sensorimotor and frontal electrodes (using wavelet transform see Fig. 2, for the same analysis using the Welch's method, see Suppl. fig. 1). According to the regression analysis, the power at the theta band was related to smartphone behavior – such that the larger the number of interactions in a minute bin, the higher the power in the corresponding bin (using wavelet transform see Fig. 2, for Welch's method, see Suppl. fig. 1). The statistically significant clusters occupied the frontal, central and occipital electrodes (see also Suppl. fig. 2 for the p-values). The alpha band was more sparsely related and the clusters occupied mostly the central and frontal electrodes – such that the larger the number of interactions the higher the power. This pattern of negative correlations extended to the beta band (18–30 Hz) with the clusters occupying almost all electrode locations. No significant clusters were detected for the variable capturing the recording duration (Suppl. fig. 3).

### 2.2 Aperiodic components related to smartphone behavior

**Fig. 3:**
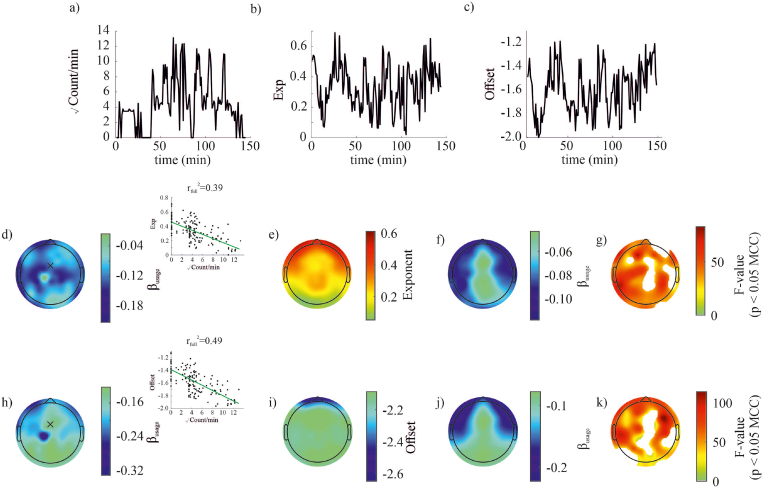
Changes of the aperiodic power-spectrum (1/f exponent) and offset with smartphone use. A reduction in the exponent and offset was found across the brain with increased smartphone use. (a) Typical smartphone use pattern throughout the experiment. Smartphone use is visualized as the square root of the number of touches in each consecutive 1 min time window. (b) The corresponding FOOOF derived 1/f exponent for each time window. (c) The corresponding FOOOF derived offset for each team window. (d) The exponent was observed to significantly reduce across the brain with increased smartphone use, as can be observed from the topography of the regression model's β-values of this individual and inset from a single electrode. (e) Grand mean topography of the exponent across time and participants. (f) Corresponding trimmed mean β-value of the regression model for the factor smartphone usage. (g) Corresponding F-values of the regression model for the factor smartphone usage masked for significance after MCC. (h–k) Legend equivalent to (d–h) but now for the offset component. The offset was observed to significantly reduce across the brain with recording time.

The aperiodic exponent was related to the number of interactions – such that the larger the number of interactions in the 1-min bin the smaller the exponent (i.e., shallower 1/f slope, using wavelet transform Fig. 3). This pattern of correlation was observed consistently at the electrodes contralateral to the (right) hand used to operate the smartphone. When using Welch's method to estimate the exponent this pattern was absent (Suppl. fig. 4). The amount of time spent in the experiment or the duration of the recording was differently related – such that the longer the time spent in the experiment the larger the exponent (i.e., steeper 1/f slope, Suppl. fig. 5 using wavelet transform - & Suppl. fig. 6 using Welch's method). This relationship was particularly prominent over the central electrodes and the contralateral parietal electrodes. The aperiodic offset – capturing the broadband shift of the periodogram – decreased with the number of smartphone interactions and increased with the time spent in the experiment (using wavelet transform: for smartphone interactions see Fig. 3 and for time see Suppl. fig. 7; using Welch's method: Suppl. fig. 4 and Suppl. fig. 8).

### 2.3 Enhanced event-related potential during periods of intense smartphone interactions

**Fig. 4:**
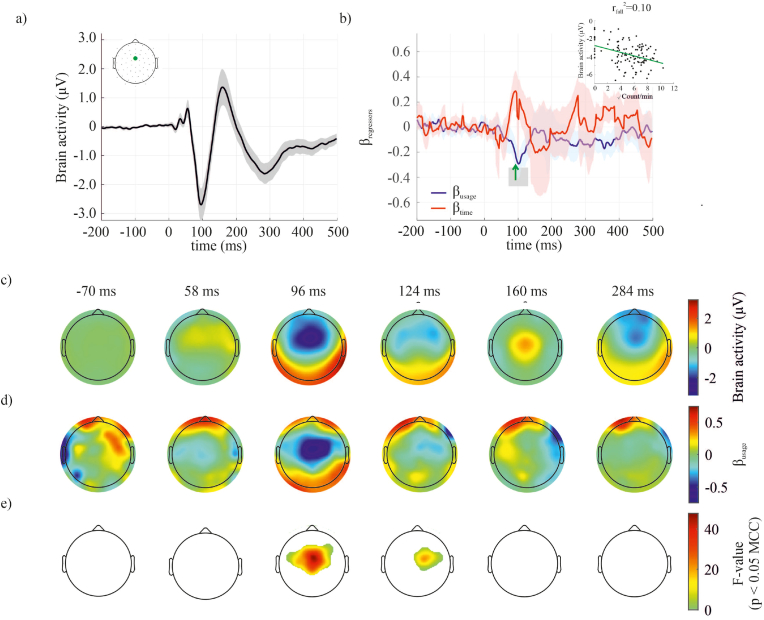
Changes in the auditory evoked potential with smartphone use and time. An enhanced peak of the auditory evoked potential at ∼100 ms was found with increased smartphone usage. (a) The mean auditory evoked potential and corresponding 95% confidence interval are shown for a midline central electrode (green dot). (b) The corresponding β-values (mean and 95% confidence interval) were derived from the regression model for the factors smartphone usage (blue line) and time (red line). The periods where significant statistical clusters related to smartphone usage were found (according to the one-sample *t*-test corrected for multiple comparison correction, MCC) are shaded in grey. The inset shows the regression model's β- value at a single time-point (for visualization). The results for the factor time can be found in Supplementary Fig. 6. (c) Scalp plots show the mean auditory evoked potential for some specific time-points. (d) Corresponding β-values of the regression model for the factor smartphone usage. (e) Corresponding F-values of the regression model for the factor smartphone usage masked for significance after MCC. (For interpretation of the references to colour in this figure legend, the reader is referred to the Web version of this article.)

The EEG signals time-locked to the auditory tones revealed significant statistical clusters as early as 58 ms at the central and frontal electrodes (see Suppl. fig. 9 and 10). Strong negativity was visible over the central electrodes at ∼100 ms followed by positivity at ∼160 ms and then again a more persistent central negativity at ∼280 ms. The event-related potentials based on the EEG data accumulated over the 1-min bins showed temporally and spatially constrained correlations to the number of smartphone interactions. The higher the number of interactions the larger (more negative) the signal amplitude at ∼100 ms over the central electrodes (Fig. 4). In the regression analysis, the time spent in the experiment (simply proportional to the number of auditory tones presented) also showed significant statistical clusters at the same latency, but the longer the duration the smaller (more positive) was the signal (see Suppl. fig. 10).

In sum, the neural oscillations, the aperiodic neural properties, and the auditory signal processing all reflected the fluctuations in smartphone interactions independently of the amount of time spent using the smartphone. These results indicate cognitive load is higher and auditory cortical processing is enhanced in periods of increased smartphone usage.

## 3 Discussion

When engaged on the smartphone, neural processing may be shaped by the momentary rise in smartphone behavioral needs. The rise in the power of theta oscillations proportional to the number of smartphone interactions indicates increased cognitive load underlay the more intense periods of smartphone usage. We further probed task-irrelevant input processing by using auditory tones. Neural processing of auditory tones was enhanced during more intense smartphone use. These findings are in keeping with the laboratory-based ideas of cognitive load theory: the higher the cognitive load the more enhanced the distractor processing.

The theta, alpha, and beta bands, and the aperiodic components were all related to increased smartphone usage. The theta band power increase was consistent with prior literature where the power reflects the extent of cognitive demands (Kahana et al., 1999). Similarly, the increase in alpha band power too was consistent with prior observations implicating it as a correlate of heightened working memory load (Jensen et al., 2002). Interestingly, the beta power was bilaterally decreased with a rise in smartphone usage. Based on the laboratory-based attempts to separate the role of alpha and beta oscillations when performing a task, the rise in alpha power signals disengagement from task-irrelevant cortical regions whereas the reduced beta-oscillations indicate reduced motor inhibition (Brinkman et al., 2014). The latter is further supported here by the concomitant decrease in the 1/f exponent (based on the wavelet transform) – which is indicative of an increased synaptic excitation:inhibition ratio (Gao et al., 2017).

The event-related potential at a latency of ∼100 ms detected over the central electrodes was specifically suppressed with the higher number of smartphone interactions. This neural signal – partly of auditory cortical origin – is considered an “exogenous” component due to its sensitivity to stimuli features: louder tones also result in larger signal amplitudes (Herrmann and Knight, 2001; Näätänen and Picton, 1987). Still, the auditory evoked potential is well implicated in attentional processes. For instance, the signal is enhanced under conditions of high visual working memory load (Regenbogen et al., 2012). Moreover, attending to auditory tones too increases the same component (Folyi et al., 2012). Taken together, it is likely that the enhanced neural signals reflect a form of sensory gain under the putatively high cognitive load induced by smartphone usage.

Our regression model also considered the time spent using the smartphone (i.e., the time spent in the recording session). The time spent was not related to the periodic components of the neural signals. However, the aperiodic exponent increased (became more negative) over time as if indicating a rise in synaptic inhibition over time according to the excitation:inhibition framework (Gao et al., 2017). Moreover, the aperiodic offset increased over time. Taken together with the findings on how the aperiodic components may reflect excitability and consciousness/arousal, these findings suggest that as time lapsed on the smartphone the underlying neural state was more inhibited (Colombo et al., 2019). The auditory event-related potential (at ∼100 ms) was also diminished with time, but as the time spent was directly proportional to the number of experienced tones, the diminished signals could be due to sensory adaption rather than the underlying putative neural relaxation.

The use of aperiodic components to infer neural states is an emerging approach, and our study provides some tangential insights into this. Firstly, the topology of the exponent is of general interest and was initially reported to follow a simple posterior to the anterior gradient at rest (Donoghue et al., 2020). However, we did not observe such a gradient either using the continuous wavelet transform or using Welch's method. The exponent was higher at the frontal electrodes, to become lower in the more posterior electrodes, and then increased again over the central electrodes akin to what has been reported in young adults (Thuwal et al., 2021). Another factor (apart from age of the participants) that may have contributed to the distinct gradient compared to the initial report is that our measurements were conducted while actively engaged in a task, and the exponent is known to alter topology in a task-dependent manner (Waschke et al., 2021). Secondly, although the wavelet transform and Welch's methods yielded similar topologies and behavioral correlates for the offset, there were notable differences. The exponents were higher for the Welch's method and the smartphone use fluctuations were related to the wavelet-derived exponents but not for the Welch's method. We chose the wavelet transform as we were interested in the time-variant spectral properties and the binning to relate with behavior could be performed after the time-frequency transformation. Welch's method – although prevalent in prior work – had to be applied to the temporally binned data which is commonly considered to be a less sound method for determining time-frequency properties. While we cannot explain the reasons underlying the differences they do demonstrate that the methodology underlying the periodogram may partly dictate the aperiodic components and their association with behavior.

So why is the neural auditory processing enhanced with rising cognitive load putatively induced by smartphone usage? In keeping with the (cognitive) load theory, one possibility is that frontal or executive functions are pre-occupied in periods of intense smartphone usage failing to effectively inhibit distractors (Lavie, 2010). This is indirectly supported by the decrease in the aperiodic offset with increased smartphone usage; reduced offset is associated with increased hemodynamic activity in the pre-frontal cortex (Jacob et al., 2021). Our findings raise further possible avenues of explanation. As indicated based on the aperiodic spectral components, when intensely interacting with the smartphone neural synaptic excitation: inhibition balance may shift towards excitation. Moreover, the enhancement could stem from increased emphasis on local computations as signaled by the rising alpha band power. All of these possibilities could well be causally interlinked. The results also raise a fundamental question on the functional relevance of enhanced processing under high load: is the enhanced processing a signature of a failing system or does it serve the purpose of making available environmental auditory inputs when otherwise deeply engaged on the smartphone? In general, our findings urge a reconsideration of how distractors are processed when engaged in real-world behavior as they may not follow common intuition.

## 4 Methods

### 4.1 Participants

Twenty-nine participants were recruited for this study from the Leiden University student community by using advertisements on flyers and a study platform. The participants were between 18 and 27 years of age (19 females). Only right-handed users with a personal Android operating smartphone were included in this study. Participants with any known neurological or mental illness, as disclosed by self-reports, were not included in the study. All experimental procedures were approved by the local ethics committee at the institute of psychology at Leiden University. Written informed consent was provided by all participants. The measurements were part of a larger study designed to address the neural correlates of smartphone behavior.

### 4.2 Smartphone data gathering

The TapCounter App (QuantActions, Lausanne) was installed on the participants' smartphones to record the timing of the touchscreen interactions at least 3 weeks before the laboratory visit. The time stamps were recorded in UTC millisecond format and processed using the scripts and download tool provided via taps.ai (QuantActions) (Balerna and Ghosh, 2018). Based on this data the four highly used – nonvideo streaming – apps were listed for laboratory use. The EEG recordings timestamps derived from a PC clock were converted to UTC millisecond format to align with the smartphone recordings. According to a separate analysis conducted in the laboratory, the mismatch between the two clocks was <4 s in a sampled population (absolute error 90th percentile 3.2 s, median 1.3 s) (Kock et al., 2022). Our subsequent analysis involved simultaneously characterizing the behavior and EEG recordings in 1 min bins. The putative clock mismatch errors mean that an EEG bin of 60 s may include <4 s of behavioral data from the neighboring bin. In each of the 1-min bins, we quantified the number of interactions. The features extracted from the EEG recordings are described below. The bin size of 1-min was driven by capturing ∼30 trials of auditory stimulations per bin (for the stimulation rate see below). Although this targeted number of trials is lower than in auditory studies that use >1000 trials, meaningful results have been obtained with ∼30 trials per block in prior studies (Alain et al., 2010; Sánchez-Morla et al., 2008).

### 4.3 EEG recording setup

EEG recordings were obtained using a 64-channel LiveAmp system (Brain Products GmbH, Gilching, Germany) together with a passive electrode cap (EasyCap, Herrsching, Germany). Sixty-two electrodes were equidistantly distributed over the scalp, and two electrodes were used to record ocular activity. All signals were online referenced against the vertex electrode and digitized at 1000 Hz. The skin was degreased by using alcohol, and the electrodes were brought in contact with the skin using Abralyt HiCl gel (EasyCap). Impedances were reduced to under 5 kOhm by pressing the gel over the skin. The system was provided supplementary power by using a power bank, and the setup, including the auditory tone generators mentioned below, was packaged into a pouch worn by the user. Short (<45 min) laboratory-based recordings were conducted in addition to the longer (∼1.5 h) recordings out of the laboratory. Only the out-of-laboratory recordings were used in the analysis. Two participants were eliminated due to data recording errors (reducing the available data from 29 to 27 individuals).

### 4.4 Auditory tones

Auditory tones were delivered by using earphones placed on both ears. A 1000 Hz tone with a pulse width of 50 ms was played at an interstimulus interval of 1–3 s (uniformly distributed). The same sequence of auditory stimuli was repeated every ∼10 min. The auditory tones were generated using an audio file stored on a smartphone. A copy of the tone was passed through an Arduino Leonardo analog to TTL converter. The TTL pulses were registered on the LiveAmp.

### 4.5 Out of the laboratory measurements

The EEG was set up within the laboratory including the initial impedance measurements. Participants were brought outside the laboratory attached to the EEG recording equipment described above and seated upright in the central entrance hallway or the main faculty restaurant. In both environments, participants used their phones for ∼45 min, after which the participant was made to switch locations. Ambient sound levels were monitored throughout the recordings using a smartphone app and microphone. The participants were encouraged to switch the app (to another pre-selected app) every 10 min by using a timer tone.

### 4.6 EEG data analysis

The data was processed and putative artifacts removed by using EEGLAB (implemented in MATLAB) (Delorme and Makeig, 2004). Channels with impedance >10 kOhm were eliminated from further consideration. The data was bandpass filtered between 0.1 and 70 Hz using a hamming windowed FIR filter (*pop_eegfiltnew* as implemented in MATLAB). Independent component analysis was run on the full recording period and the blink-related components were automatically detected and removed (Pontifex et al., 2017). Subsequently, the data were filtered with a low pass 45 Hz filter (for ERP analysis) and the eliminated channels were interpolated. The data were re-referenced to the common average of the scalp electrodes.

Towards the event-related potential analysis, the data were seperated in 1-min bins. At each bin, the data was epoched spanning −200 to 500 ms from the stimulation. The baseline was removed based on the signal mean from – 200 to 0 ms for each epoch. Epochs at the bin edges were eliminated. A minimum of 10 stimulations were necessary for inclusion in further analysis. The event-related potential was based on trimmed (truncated) means (20% as implemented in LIMO EEG toolbox, function *limo_trimci*) (Pernet et al., 2011).

Towards the frequency analysis, the continuous wavelet transform was estimated for each channel and over the entire recording period using the function *cwt* (implemented in MATLAB, MathWorks). Towards this, 30 voices per octave and a time-bandwidth of 120 were used over a frequency range of 0.1–40 Hz. The transformed data were then binned over 1 min windows to estimate the periodogram based on the squared mean magnitudes across the time window. In a parallel set of analyses, we used Welch's method for spectral analysis using the function *pwelch* (implemented in MATLAB). The periodic and aperiodic components were separated by using the FOOOF toolbox (Donoghue et al., 2020). For both the event-related potential and the frequency analysis, the square root of the total number of interactions in the given bin, and the time elapsed at the bin onset were recorded for subsequent analysis.

### 4.7 Statistical analysis

We performed hierarchical linear modeling by using LIMO EEG across all electrodes, and time points or frequencies (including aperiodic components). As mentioned above, the continuous data were binned into 1-min bins to extract four types of parameters from each bin: (i) the event-related potential, (ii) the spectral properties including the periodogram and aperiodic components, and (iii) the time spent in the recording along with (iv) the number of interactions. At the first level of analysis, we performed a mass univariate regression (iterative least squares) with the recording time elapsed and the square root of the number of interactions as explanatory variables. A threshold of 10 bins was set for this step (i.e., a minimum of 10, 1-min, bins were necessary to proceed with the regression). The regression slopes (*β*_time_ and *β*_usage_) were then gathered across all participants (with a standard deviation of the smartphone usage variable >1). At the second level, one-sample *t*-tests were performed (using trimmed means) across all electrodes and time points or frequencies and then corrected for multiple comparisons correction. For multiple comparison correction, two-dimensional (spatial-temporal or spatial-frequency) clustering was based on 1000 bootstraps (α = 0.05). Three of the 27 subjects were eliminated due to inexplicable signals spotted during visual inspection of the data, which were probably a result of poor grounding or electrode bridges during the recording. For the ERP analysis, 23 subjects had the sufficient number of trials and were analyzed further. For the spectral analysis, all of the 24 subjects were included.

## Data availability

The data used in this report is made available on dataverse.nl within 1 month of publication according to the guidelines of the Institute of Psychology, Leiden University.

## Code availability

The codes used in this report are made available on GitHub (https://github.com/CODELABLEIDEN/Audio_tone_Smartphone_Outdoor_2022). The software dependencies are mentioned above.

## Author contributions

MR and AG conceptualized the study. MR gathered the data aided by AG. AG analyzed the data, and MR visualized the analysis. AG drafted the report aided by MR.

## Declaration of competing interest

AG is a co-founder of QuantActions Ltd., Lausanne, Switzerland. This company focuses on converting smartphone taps to mental health indicators. Software and data collection services from QuantActions were used to monitor smartphone activity.
