# Supplementary material for: Can you hear me now? Momentary increase in smartphone usage enhances neural processing of task-irrelevant sound tones
Source: Neuroimage Rep. 2022 Sep 13;2(4):100131. doi: 10.1016/j.ynirp.2022.100131 (PMC12172703; doi:10.1016/j.ynirp.2022.100131)
Supplement: Multimedia component 1 [file mmc1.pdf]

Can you hear me now? Momentary increase in smartphone usage enhances neural processing of task-irrelevant sound tones

Mark van de Ruit and Arko Ghosh

Supplementary Figures

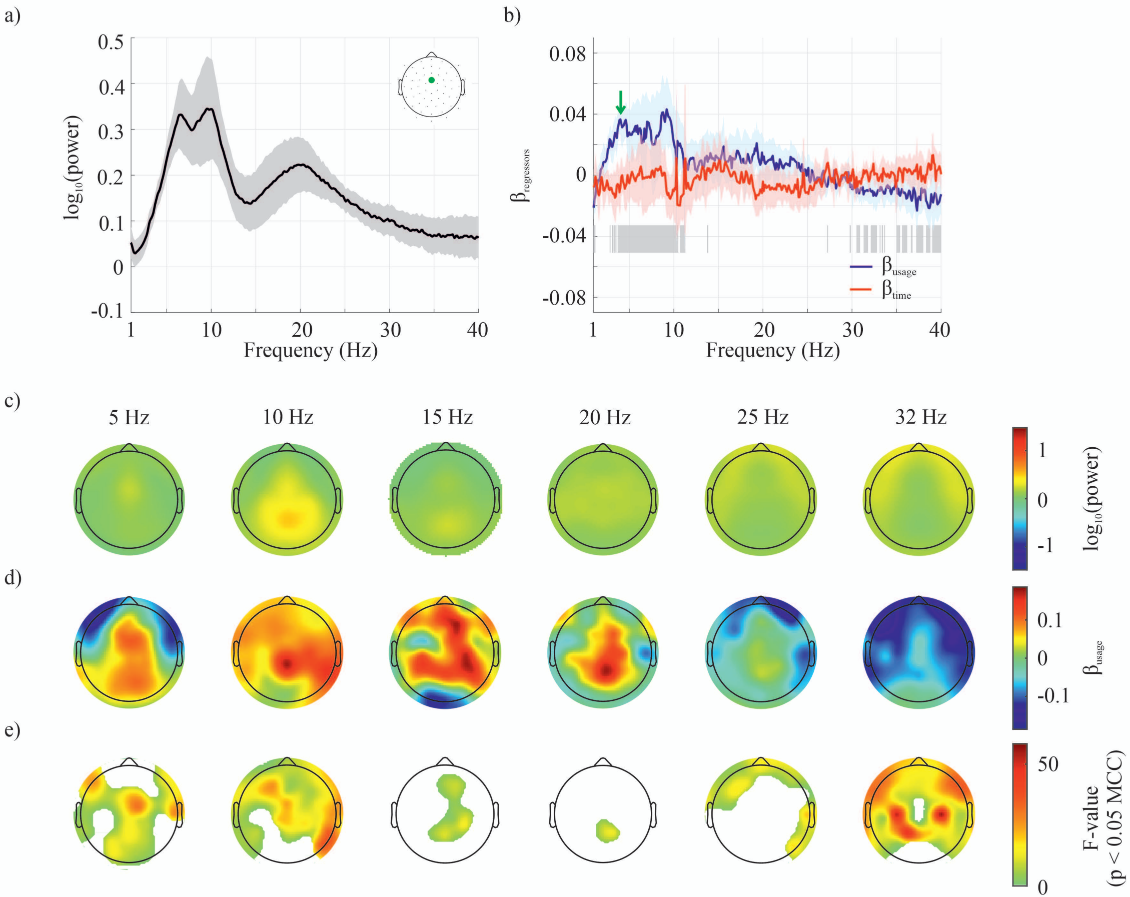

a)
0.5
0.4
0.3
0.2
0.1
0
-0.1
log10(power)
1
10
20
30
40
Frequency (Hz)
b)
0.08
0.04
0
-0.04
-0.08
βregressors
βusage
βtime
1
10
20
30
40
Frequency (Hz)
c)
5 Hz
10 Hz
15 Hz
20 Hz
25 Hz
32 Hz
1
0
-1
log10(power)
d)
0.1
0
-0.1
βusage
e)
50
0
F-value
(p < 0.05 MCC)

Supplementary figure 1: EEG power spectrum, determined using Welch's methods, is related with smartphone usage and the time spent on the phone. The correlations were observed at the level of each subject using linear regressions, and we performed one-sample t-tests of the regression coefficients (β) stemming from each subject. An increase in central theta band power (4-8 Hz) is observed with increased smartphone usage. Legend equivalent to Fig 2 (a-e).

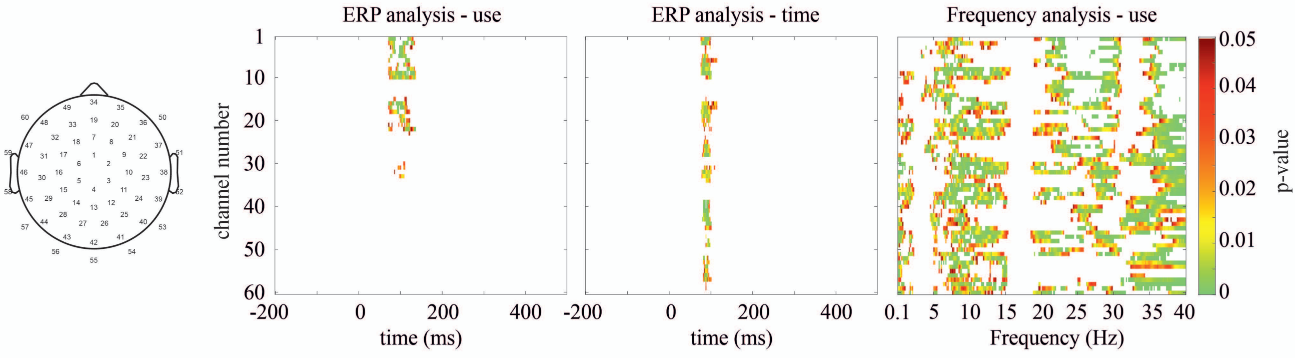

ERP analysis - use
ERP analysis - time
Frequency analysis - use
channel number
time (ms)
time (ms)
Frequency (Hz)
p-value

Supplementary figure 2: P-values for significant clusters and all channels for one-sample t-tests of β-values. (From left to right) AEP analysis for factor smartphone usage, AEP analysis for factor time and frequency analysis for factor smartphone usage.

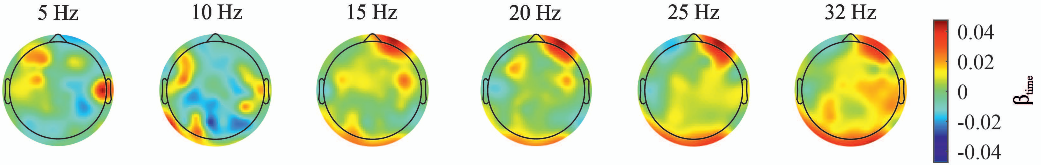

5 Hz
10 Hz
15 Hz
20 Hz
25 Hz
32 Hz
0.04
0.02
0
-0.02
-0.04
β_{time}

Supplementary figure 3: Scalp plots for the β-values of the regression model for the factor time against EEG power. No significant β-values were found at any frequency.

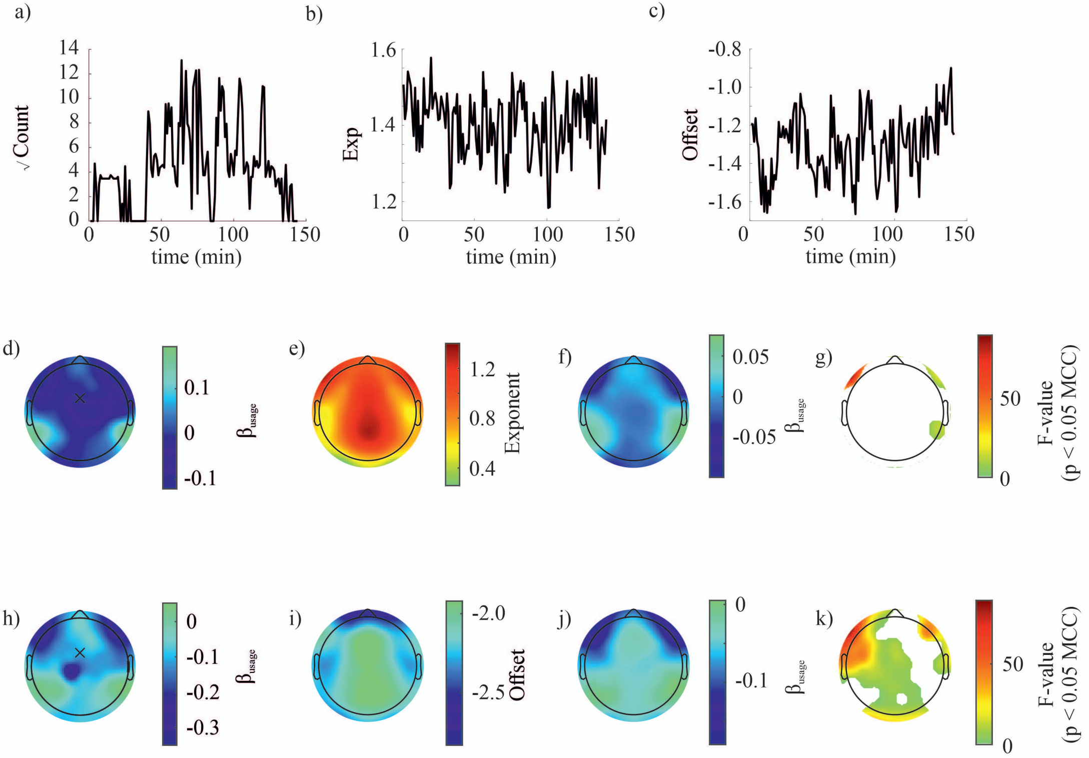

a)
b)
c)
√Count
Exp
Offset
time (min)
time (min)
time (min)
d)
e)
f)
g)
β_{usage}
Exponent
β_{usage}
F-value
(p < 0.05 MCC)
h)
i)
j)
k)
β_{usage}
Offset
β_{usage}
F-value
(p < 0.05 MCC)

Supplementary figure 4: Changes of the aperiodic Welch's method power-spectrum (1/f exponent) and offset with smartphone use. A significant reduction in the offset, but not the exponent, was found across the brain with increased smartphone use. Individual data represents the same participant as in Fig. 3. Legend equivalent to Fig. 3 (a-k).

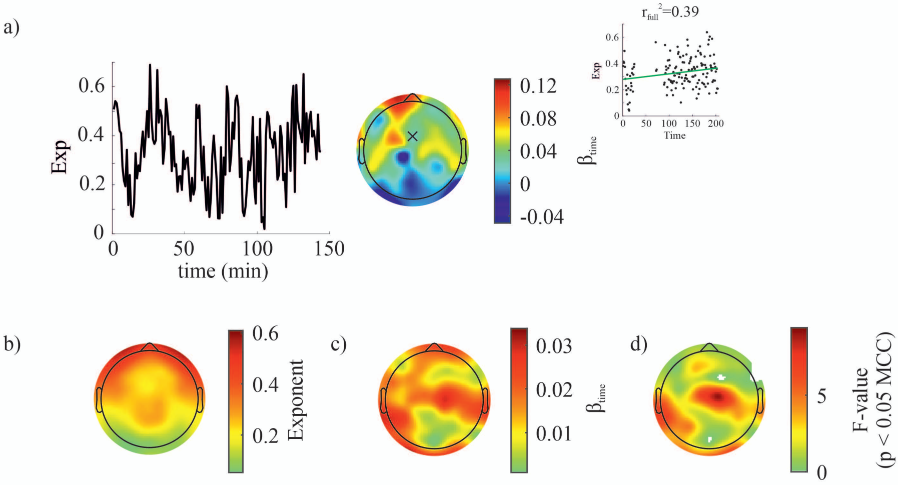
a)
Exp
0.6
0.4
0.2
0
0
50
100
150
time (min)
0.12
0.08
0.04
0
-0.04
βtime
rfull2=0.39
Exp
0.6
0.4
0.2
0
0
50
100
150
200
Time
b)
0.6
0.4
0.2
Exponent
c)
0.03
0.02
0.01
βtime
d)
5
0
F-value
(p < 0.05 MCC)

Supplementary figure 5: Changes of the FOOOF derived aperiodic exponent with time. A significant increase in the exponent is found across the brain with increased time in the experiment. Individual data represents the same participant as in Fig. 3. Legend equivalent to Fig. 3 (b, d-g).

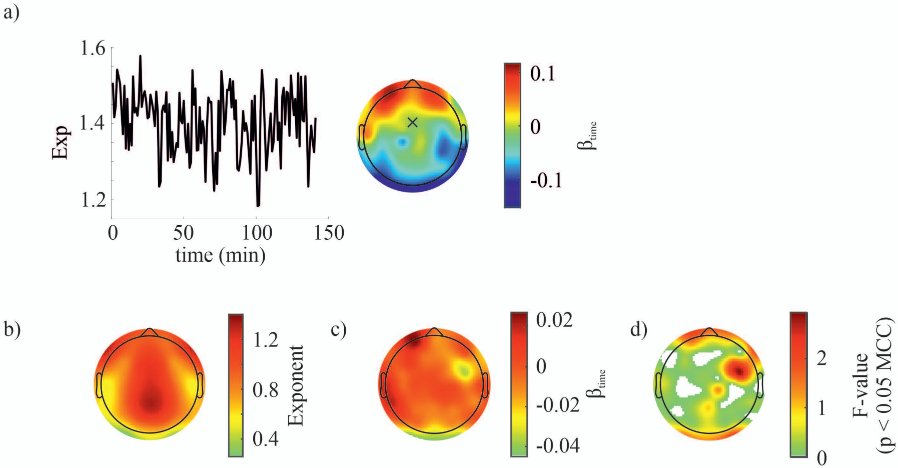
a)
1.6
1.4
1.2
Exp
0
50
100
150
time (min)
0.1
0
-0.1
β_{time}
b)
1.2
0.8
0.4
Exponent
c)
0.02
0
-0.02
-0.04
β_{time}
d)
2
1
0
F-value
(p < 0.05 MCC)

Supplementary figure 6: Changes of the FOOOF derived Welch's method aperiodic exponent with time. A significant increase in the exponent is found across the brain with increased time in the experiment. Individual data represents the same participant as in Fig. 3. Legend equivalent to Fig. 3 (b, d-g).

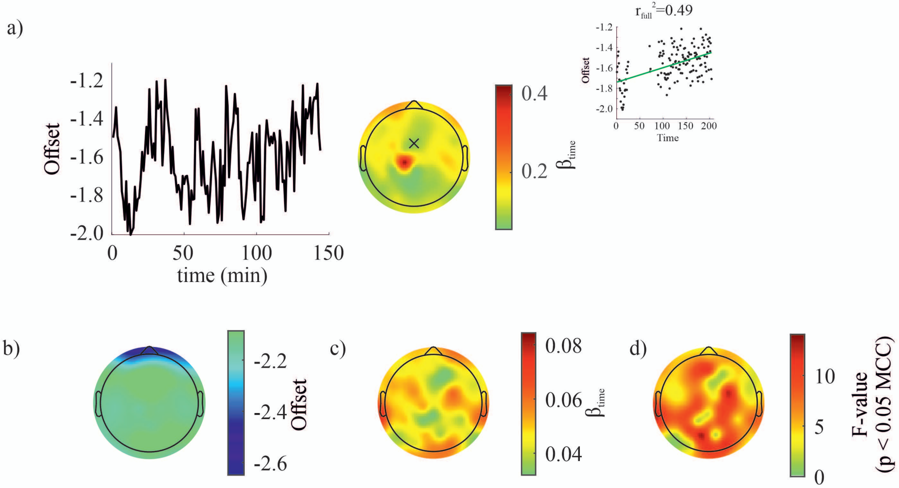

a)
b)
c)
d)
Offset
-1.2
-1.4
-1.6
-1.8
-2.0
0
50
100
150
time (min)
0.4
0.2
β_{time}
r_{full}^2=0.49
Offset
Time
200
-2.2
-2.4
-2.6
Offset
0.08
0.06
0.04
β_{time}
10
5
0
F-value
(p < 0.05 MCC)

Supplementary figure 7: Changes of the FOOOF offset with time. A significant increase in the offset is found across the brain with time in the experiment. Individual data represents the same participant as in Fig. 3. Legend equivalent to Fig. 3 (b,d-g).

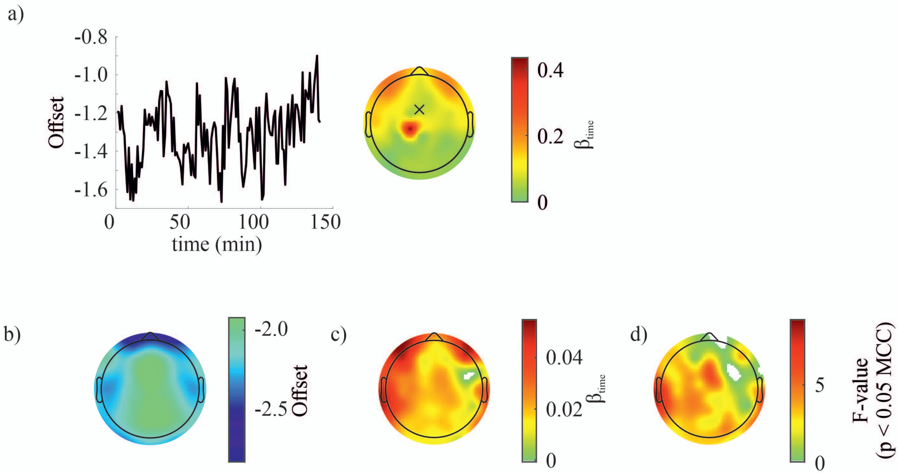

a)
-0.8
-1.0
-1.2
-1.4
-1.6
Offset
0
50
100
150
time (min)
0.4
0.2
0
β_{time}
b)
-2.0
-2.5
Offset
c)
0.04
0.02
0
β_{time}
d)
5
0
F-value
($p < 0.05$ MCC)

Supplementary figure 8: Changes of the FOOOF Welch's method offset with time. A significant increase in the offset is found across the brain with time in the experiment. Individual data represents the same participant as in Fig. 3. Legend equivalent to Fig. 3 (b,d-g).

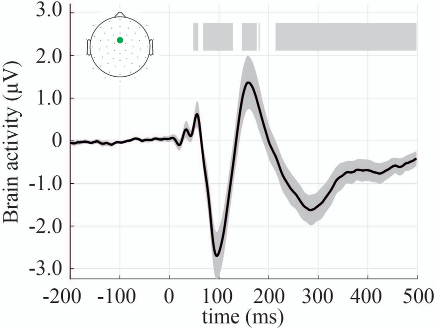
3.0
2.0
1.0
0
-1.0
-2.0
-3.0
Brain activity (μV)
-200
-100
0
100
200
300
400
500
time (ms)

Supplementary figure 9: Significance of the auditory evoked potential compared to zero, highlighting the peak activity around ~50, 100, 150 and 300 ms.

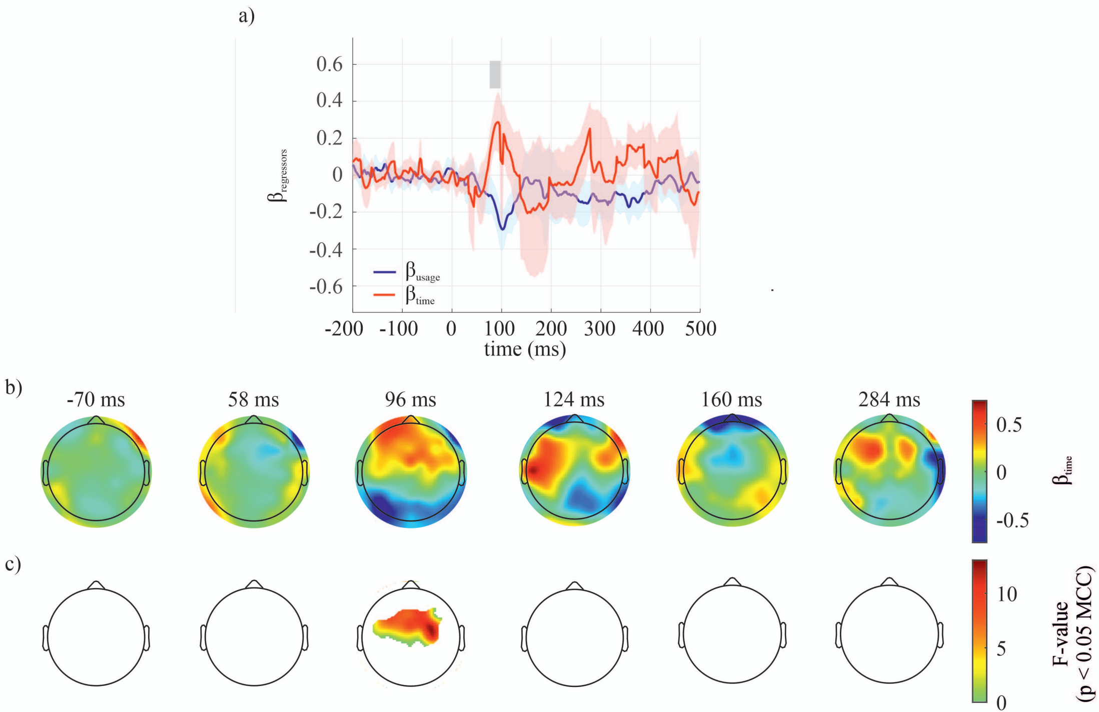

a)
0.6
0.4
0.2
0
-0.2
-0.4
-0.6
β_regressors
β_usage
β_time
-200
-100
0
100
200
300
400
500
time (ms)
b)
-70 ms
58 ms
96 ms
124 ms
160 ms
284 ms
0.5
0
-0.5
β_time
c)
10
5
0
F-value
(p < 0.05 MCC)

Supplementary figure 10: (a) β-values (mean and 95% confidence interval) derived from the regression model for the factors smartphone usage (blue line) and time (red line) (same as Figure 4b). The periods where significant effects of time on the AEP are found (according to one sample t-test corrected for multiple comparison correction, abbreviated as MCC) are shaded in grey. (b) Scalp plots of β-values of the regression model for the factor time. (c) Corresponding F-values of the regression model for the factor time masked for significance after MCC.
